# Genome-wide association study and pathway analysis to decipher loci associated with Fusarium ear rot resistance in tropical maize germplasm

**DOI:** 10.1007/s10722-023-01793-4

**Published:** 2023-11-10

**Authors:** Stella Bigirwa Ayesiga, Patrick Rubaihayo, Bonny Michael Oloka, Isaac Ozinga Dramadri, Julius Pyton Sserumaga

**Affiliations:** 1 Department of Agricultural Production, College of Agriculture and Environmental Sciences, Makerere University, P. O. Box 7062, Kampala, Uganda; 2 Department of Horticultural Sciences, North Carolina State University, Raleigh, NC USA; 3 National Livestock Resources Research Institute, National Agricultural Research Organization, PO Box 5704, Kampala, Uganda

**Keywords:** Genome-wide association study, Metabolic pathways, *Fusarium verticillioides*, SNPs, *Zea mays*

## Abstract

Breeding for host resistance is the most efficient and environmentally safe method to curb the spread of fusarium ear rot (FER). However, conventional breeding for resistance to FER is hampered by the complex polygenic nature of this trait, which is highly influenced by environmental conditions. This study aimed to identify genomic regions, single nucleotide polymorphisms (SNPs), and putative candidate genes associated with FER resistance as well as candidate metabolic pathways and pathway genes involved in it. A panel of 151 tropical inbred maize lines were used to assess the genetic architecture of FER resistance over two seasons. During the study period, seven SNPs associated with FER resistance were identified on chromosomes 1, 2, 4, 5, and 9, accounting for 4–11% of the phenotypic variance. These significant markers were annotated into four genes. Seven significant metabolic pathways involved in FER resistance were identified using the Pathway Association Study Tool, the most significant being the superpathway of the glyoxylate cycle. Overall, this study confirmed that resistance to FER is indeed a complex mechanism controlled by several small to medium-effect loci. Our findings may contribute to fast-tracking the efforts to develop disease-resistant maize lines through marker-assisted selection.

**Supplementary Information:**

The online version contains supplementary material available at 10.1007/s10722-023-01793-4.

## Introduction

Fusarium ear rot (FER), which is caused by *Fusarium verticillioides* (Saccardo) Nirenberg, is a significant disease that affects maize worldwide (Stagnati et al. 2019) targeting almost all of its growth stages (Lanubile et al. 2014). FER leads to significant yield losses, which are estimated between 10 and 30% and can reach 50% or more in severely infected regions (Yao et al. 2020). In addition, this disease leads to poor grain quality and contamination of the infected kernels with fumonisin, a mycotoxin and known carcinogen reported to be harmful to both animal and human health (Czembor et al. 2019; Stagnati et al. 2019). In areas where maize is a staple food, such as sub-Saharan Africa, FER infection has been reported to be high (Bigirwa et al. 2007; Tembo et al. 2022).

Traditional FER management methods primarily involve the use of fungicides or other agronomic approaches, but these have been reported to be ineffective and environmentally unfriendly, and to increase the costs of maize production (Lanubile et al. 2017). Breeding for disease resistance is recommended because it is an efficient and ecologically safe strategy (Chen et al. 2016; Lanubile et al. 2017). Despite the benefits of using resistant cultivars, only a few resistant genotypes are available because of the complex genetic architecture of FER resistance (de Jong et al. 2018). This complexity is attributed to the fact that the resistance mechanism is controlled by multiple genes with minor effects that are highly influenced by the environment and are not consistent between populations (Butrón et al. 2015; Chen et al. 2012; Clements et al. 2004; de Jong et al. 2018; Holland et al. 2020; Samayoa et al. 2019; Zila et al. 2013).

Genome-Wide Association Studies (GWASs) are particularly suitable for the identification of marker-trait associations in complex quantitative traits using diverse germplasm lines (Cui et al. 2016; Samayoa et al. 2019). GWASs based on genetic linkage disequilibrium (LD) are preferred to traditional linkage-based analyses because of their excellent mapping resolution that allows to capture and map small effect loci (Sitonik et al. 2019). In maize, GWASs have successfully been used to detect genomic regions (Chen et al. 2016; Wang et al. 2012; Zila et al. 2013, 2014) and analyze the genetic architecture of various important and complex traits, such as resistance to aflatoxin and ear rot caused by *Aspergillus flavus* (Tang et al. 2015; Warburton et al. 2015), common maize rust caused by *Puccinia sorghi* Schwein (Kibe et al. 2020; Olukolu et al. 2016), northern corn leaf blight (Ding et al. 2015; Rashid et al. 2020), oil biosynthesis (Li et al. 2013), resistance to head smut (Wang et al. 2012), and seedling root development (Pace et al. 2015).

In addition to identifying genomic regions and genes involved in disease resistance, GWASs also assist in identifying resistance pathways and associated genes. Metabolic pathway analysis focuses on the combined effects of many genes clustered together because of their shared biological functions (Tang et al. 2015; Warburton et al. 2022). This type of research complements the study of the most significant marker-trait associations in addition to giving clues on the genetic basis of specific traits (Tang et al. 2015). Combining FER resistance data derived from GWASs in a pathway analysis allows to jointly consider all the genetic sequences positively associated with resistance to this disease and consequently to potentially identify pathways and associated genes involved in it. Identifying these genes will eventually lead to more efficient breeding procedures and the development of FER-resistant maize hybrids. A better understanding of the pathways involved in resistance will also lead to a broader understanding of plant defense mechanisms against other fungi.

The aim of this study was to identify genomic regions, single nucleotide polymorphisms (SNP), and putative candidate genes as well as candidate metabolic pathways and associated genes involved in FER resistance.

## Materials and methods

### Plant materials and field management

A total of 151 inbred maize lines were evaluated at the National Livestock Resources Research Institute (NaLIRRI) of the National Agricultural Research Organization of Uganda, which is located in a mid-altitude agroecological zone (0° 32’N and 32° 35´E) at 1150 m above sea level (Sserumaga et al. 2021). Detailed information on these inbred lines is included in our previous study (Ayesiga et al. 2023). The Alpha lattice design with two replications was used in the present study. The two-row plots were 5 m long and placed 0.75 m apart, and the spacing between plants was 0.5 m. Two seeds per hill were planted and, 4 weeks after seedling emergence, one of them on each hill was removed. Standard agronomic and cultural practices were followed. Fertilizers were applied at two different rates: 77 kg N ha^−1^ at planting and 27 kg P ha^−1^ at topdressing 4 weeks after planting. Phenotyping for FER was conducted over two seasons.

### FER inoculation and evaluation

**Fig. 1:**
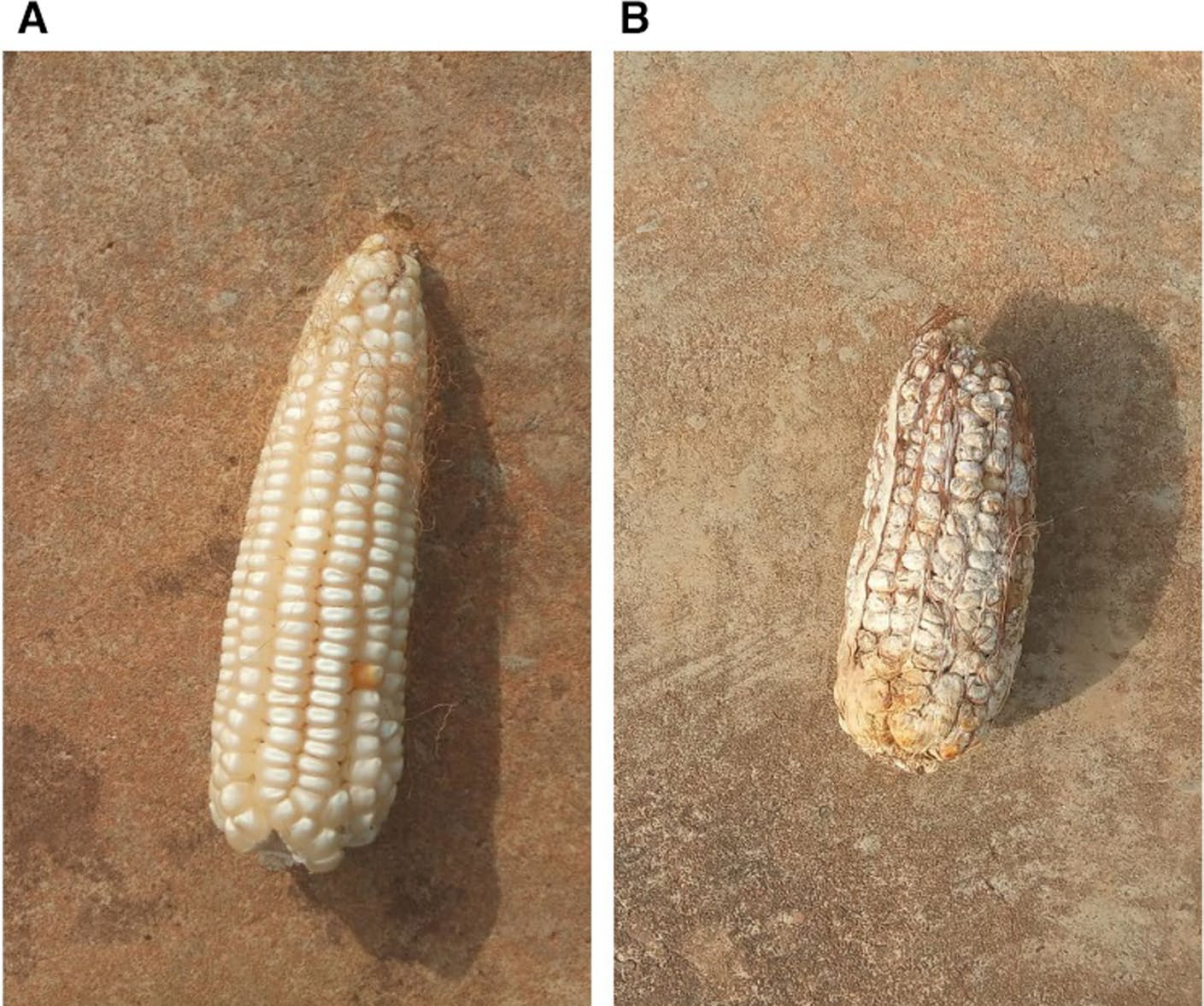
Images of maize showing fusarium ear rot symptoms scored as 1 **A** and 9 **B** on the nine-point scale

The pathogen was initially isolated from infected maize cobs obtained from the fields at NaLIRRI. The inoculum was prepared using a procedure modified from Chambers (1988). The infected grains were sterilized for 3 min in 10% commercial JIK bleach containing 0.39% sodium hypochlorite (NaClO) solution (Tembo et al. 2013) and then rinsed three times using distilled water. The sterilized infected grains were then placed in a flask together with toothpicks. Before use, the toothpicks were autoclaved to remove tannins and other antifungal compounds. The flask containing the infected grains and toothpicks was then sealed and left standing for 3 weeks to allow the fungus to grow on the toothpicks. The fully colonized toothpicks were used to inoculate the maize ears approximately 7 days after flowering. Inoculation was conducted by piercing through the middle of the primary ear of five plants per plot. Paper bags were used to cover the ears to avoid allo-infection. At maturity, the inoculated ears from each plot were harvested, and FER symptoms were assessed based on the percentage of infected area using the following nine-point scale: 1 = 0% (no visible disease symptom), 2 = 1%, 3 = 2–5%, 4 = 6–10%, 5 = 11–20%, 6 = 21–40%, 7 = 41–60%, 8 = 61–80%, and 9 = 81–100% (Fig. 1) (Guo et al. 2020).

### DNA isolation and genotyping

Once the three-leaf stage was reached, leaf samples from all the 151 inbred lines were harvested, packaged, and shipped for DNA extraction and genotyping at the Integrated Genotyping Sequence Support (IGSS) at the Bioscience for East and Central Africa (BecA)-Hub in Nairobi, Kenya. Imputation of the missing markers and data filtering were carried out for a minimum count of 80% of the sample size using TASSEL v.5.2 software (Bradbury et al. 2007). To avoid spurious marker-trait associations, monomorphic SNPs with missing data points > 10%, a minor allele frequency < 0.05, and heterozygosity > 5% were discarded, leaving a total of 20,900 high-quality SNPs distributed across 10 chromosomes for analysis.

### Statistical analyses

The effects of seasons and inbred lines on the severity of FER were analyzed in R using the agricolae package (R Core Team 2015). Analysis of variance (ANOVA) was also performed using the same package in R and applying the restricted maximum likelihood method based on the following equation:

$$Y{\text{ijk}} = \, \mu \, + Gi + Rj + R/B{\text{jk}} + \varepsilon {\text{ijk}}$$

where *Y*ijk is the *k*th observation for the *i*th genotype, *μ* is the overall mean, *Gi* is the genotype effect, *Rj* is the replication effect, and *R/B*jk is the effect of blocks nested in replicates; *ε*ijk is the error term associated with *Y*ijk*.* Best linear unbiased predictors were computed in R (R Core Team 2015).

### Population structure, linkage disequilibrium, and association analysis

Principal component analysis (PCA) was performed using the Genome Association and Prediction Integrated Tool (GAPIT) in R (R Core Team 2015) to assess the population structure of the 151 inbred lines. The relative kinship coefficient matrix (K) was generated to determine the relatedness among inbred lines (Liu et al. 2021) using the GAPIT package (Lipka et al. 2012). Pairwise measures of linkage disequilibrium (LD) were calculated to assess the degree of cosegregation among the blocks of SNPs in TASSEL v.5.2 (Bradbury et al. 2007). The LD decay rate between each pair of SNPs was determined based on the squared Pearson correlation coefficient (r^2^). To estimate the overall LD decay pattern over genetic distances, pairwise LD r^2^ estimates from the 10 chromosomes examined were plotted against the corresponding pairwise genetic distances in base pairs (Coan et al. 2018) using R software (R Core Team 2015).

A GWAS was conducted using the multi-locus fixed model and random circulating probability unification model in R via the rMVP package (Liu et al. 2016). After the analysis, Manhattan plots were generated to visualize the associations between SNP markers and the trait of interest (i.e., FER resistance) by plotting the genomic positions of the SNPs against their negative log base 10 of the p-values obtained from the GWAS model. The overall proportion of phenotypic variance explained by the discovered quantitative trait loci (QTLs) was obtained by fitting all significant SNPs together in a linear model to determine R^2^. The putative candidate genes containing or adjacent to the significant SNPs were identified using the B73 reference genome information in the MaizeGDB database (https://www.maizegdb.org).

### Pathways Analysis

The GWAS output was run through the Pathway Analysis Study Tool (PAST) (Thrash et al. 2020) on the MaizeGDB website (https://www.maizegdb.org/past), as described in Tang et al. (2015) and Warburton et al. (2022). The data used in PAST included the p-values (the significant SNP-trait association values); R^2^ (proportion of the explained phenotypic variation), effect values along with the calculated LD values for D’ and R^2^, and the p-value between each SNP marker and its closest neighboring SNPs (Tang et al. 2015; Warburton et al. 2022). SNPs were then assigned to genes, and the functions of candidate genes were assessed by examining the pathways in which the encoded enzymes were involved (Warburton et al. 2022). In this process, the SNP marker sequences were aligned to the B73 reference genome, and then the overlapping genes with the highest blast score and identity percentage were selected (Tang et al. 2015). The candidate genes’ gene ontology, molecular functions, and biological processes were obtained from the MaizeGDB database (https://www.maizegdb.org). For the pathway analysis, which was conducted in PAST, the SNP to gene algorithm was run for associations for the FER data across the two seasons, and genes were grouped into pathways. Several genes contributed to these pathways and were ranked according to their running enrichment score (RES). The RES shows the extent to which the genes in a gene set are overrepresented at the extremes (either top or bottom) of their complete ranked list. Only pathways with at least five annotated genes were analyzed to avoid minor sample size effects (Tang et al. 2015; Warburton et al. 2022). The genes that contributed the most to the RESs of pathways with FDR < 0.2 were selected for further analysis (Tang et al. 2015). Pathway Identification (PWID) values were assigned based on the CornCyc database (https://maizegdb.org/metabolic_pathways).

## Results

### Statistical analysis

FER severity for all the 151 inbred lines across the two seasons examined ranged from score 1 (1% ear rot symptoms) to 9 (over 80% ear rot symptoms). The ANOVA results showed significant variation (*P* < 0.05) among inbred lines; there was also considerable variation across the two seasons and in the genotype-by-season interaction. In the combined ANOVA, differences in FER severity among seasons and inbred lines were significant (Table 1). Overall, 12 inbred lines had the lowest scores for FER symptoms across the two seasons, namely, CKL150038, CKL150105, CKL150109, CKL150105, JPS25-11, JPS26-4, JPS25-40, JPS25-36, JPS25-14, JPS25-11, DL141392, and WL429-24.

**Table 1:** Combined analysis of variance for the 151 maize inbred lines evaluated after artificial *Fusarium verticillioides* inoculation at Namulonge across two seasons

Source of variation	Degrees of freedom	Mean square
Replication	1	4.86
Season	1	276.08***
Genotype	150	331.02***
Replication:Block	109	120.36
Season:Genotype	149	374.67***
Residual	192	181.78

*, **, *** Significant at the 0.05, 0.01, and 0.001 probability levels, respectively

### Population structure and LD

**Fig. 2:**
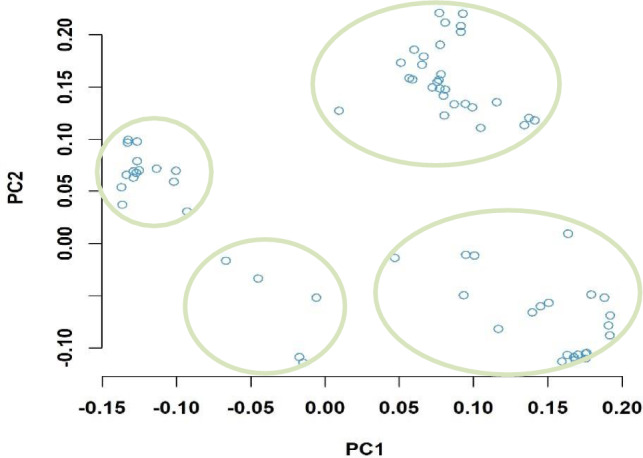
Principal component (PC) biplot showing the clustering of the 151 tropical maize inbred lines assessed in this study

**Fig. 3:**
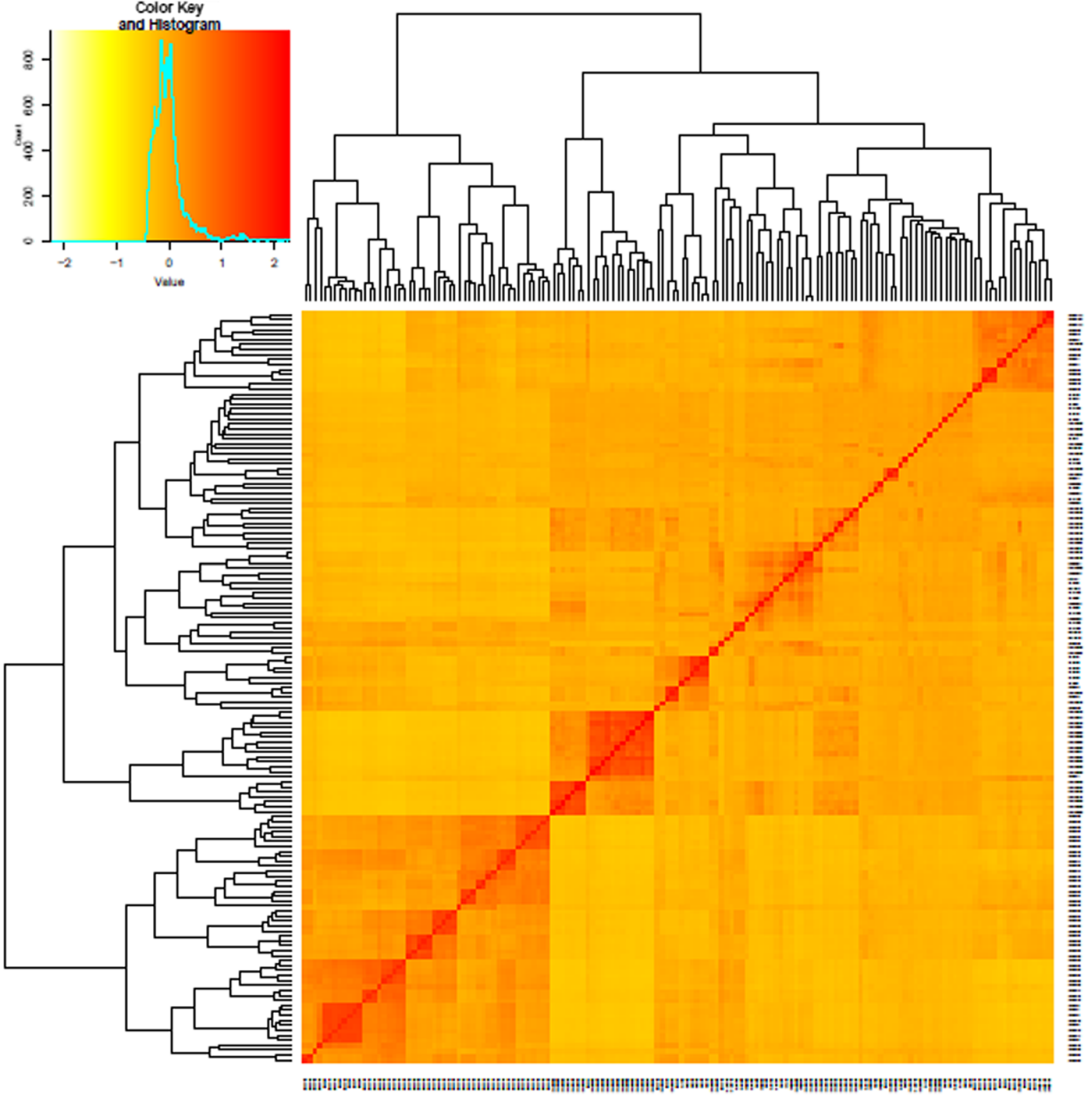
Heat map of the kinship matrix values showing the level of relatedness among inbred lines (the darker red regions indicate highly related lines)

**Fig. 4:**
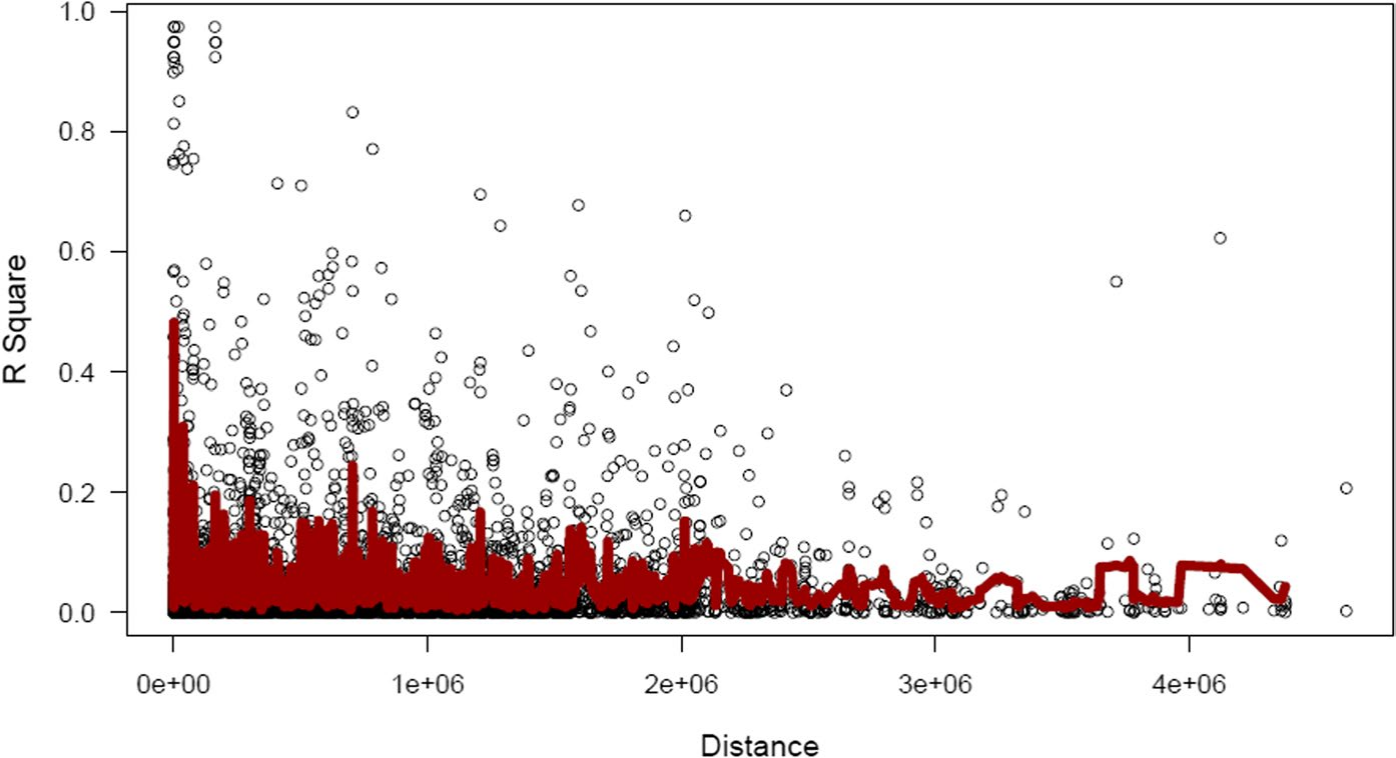
Genome-wide average linkage disequilibrium (LD) decay *r*^2^ values over genetic distances (bp) showing that LD decayed rapidly as the distance between single nucleotide polymorphisms increased

**Fig. 5:**
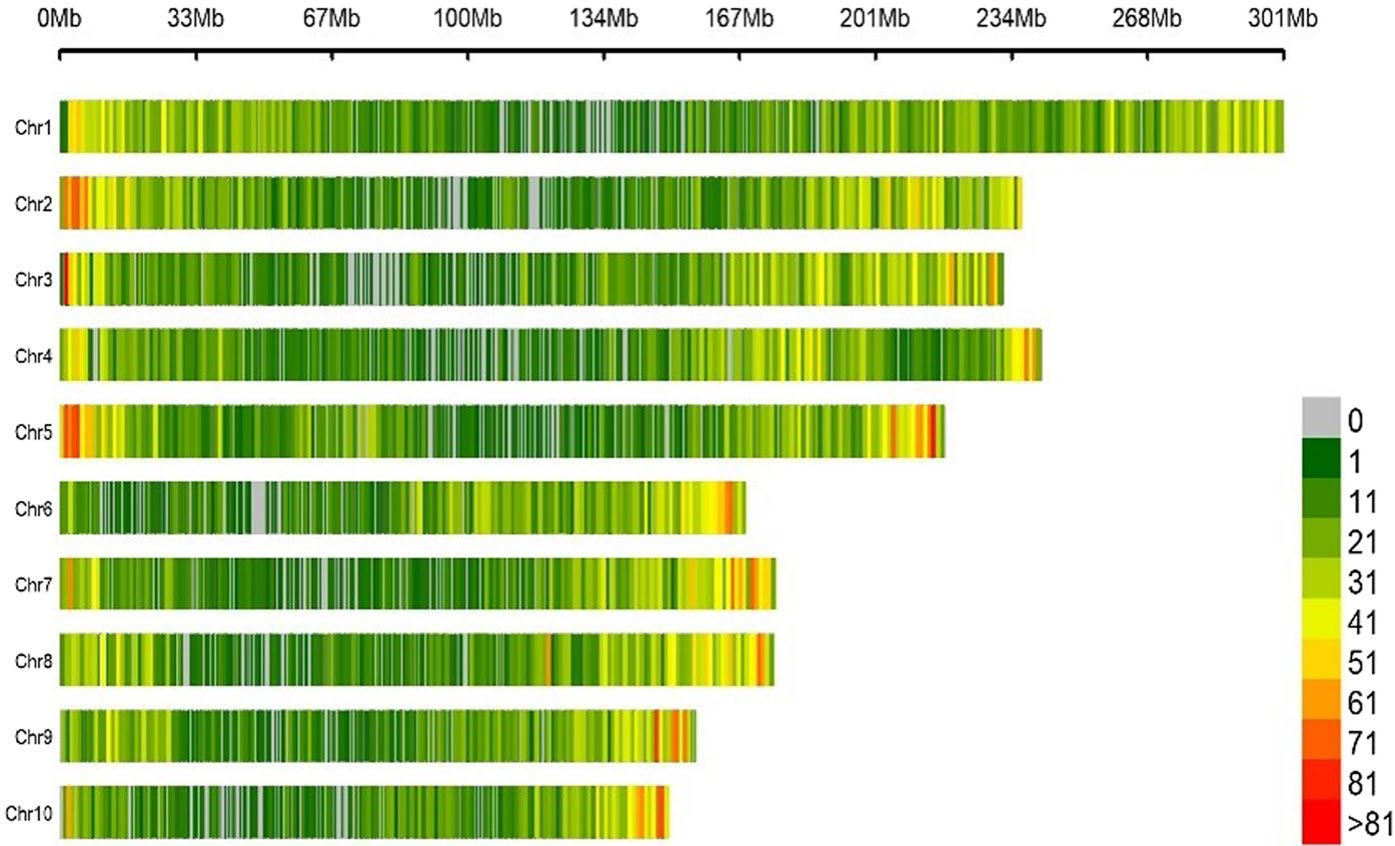
Distribution of high-quality single nucleotide polymorphisms retained for population and marker-trait analysis against the B73 reference genome

Based on the results of population structure analysis, the 151 inbred lines were divided into four subgroups. In this subdivision, 54% of the inbred lines were assigned to distinct groups and the remaining lines were assigned into a mixed group. Detailed information on these lines is included in Ayesiga et al. (2023). The grouping was confirmed using PCA, as illustrated by the first two principal components (Fig. 2) and a heatmap (Fig. 3). Both the population structure (Ayesiga et al. 2023) and the kinship matrix revealed a clear differentiation of the assessed maize inbred lines. LD analysis showed that LD declined as the distance between SNP markers increased (Fig. 4). In this study, a total of 519,417 marker pairs were detected (based on SNP combinations across 10 chromosomes), and the SNPs were uniformly distributed across the 10 chromosomes (Fig. 5).

### Association mapping

**Fig. 6:**
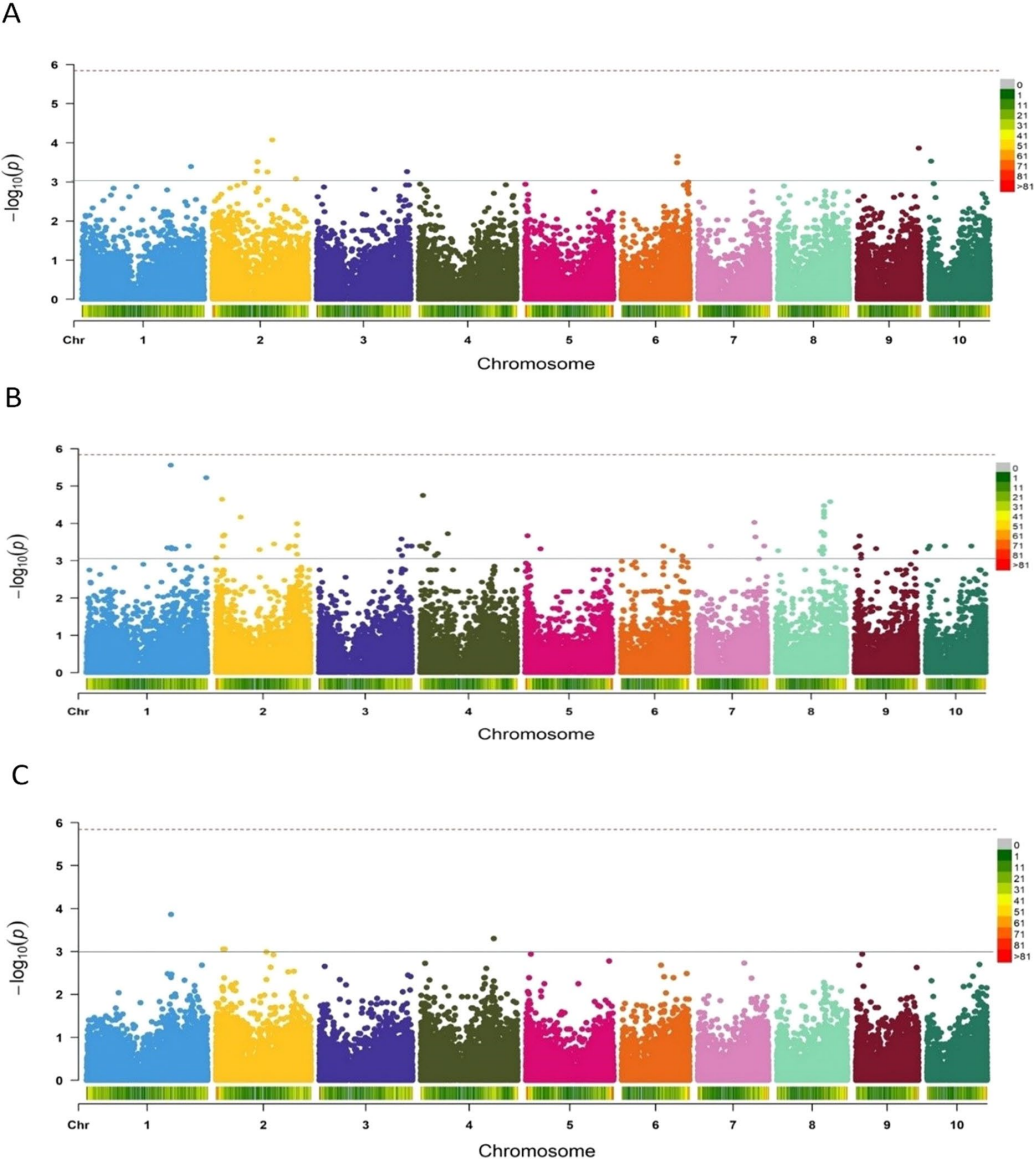
Manhattan plots for the genome wide analysis study (GWAS) of fusarium ear rot resistance showing significant single nucleotide polymorphisms (SNPs) detected in season 1 (10 SNPs) (**A**), season 2 (20 SNPs) (**B**), and combined seasons (7 SNPs) (**C**)

The GWAS conducted using data for season one identified 10 SNPs significantly associated with FER resistance at *P* < 1 × 10^−3^ on chromosomes 1, 2, 3, 6, 9, and 10 (Fig. 6A) with phenotypic variation ranging from 8.6 to 9.9%. The GWAS using the data for season two identified 20 significant SNPs on all chromosomes except for 3, 6, and 10 (Fig. 6B), and the phenotypic variation accounted for by these SNPs ranged from 8.4 to 12.3%. In the analysis combining the two seasons, seven significant SNPs were identified at P < 1 × 10^−3^ on chromosomes 1, 2, 4, 5, and 9 (Fig. 6C). Across the two seasons, the most significant SNP (2,396,181|F|0–39:G > T-39:G > T) was identified on chromosome 1, and the least significant (2,428,673|F|0–67:A > G-67:A > G) on chromosome 9. The seven SNPs detected in the abovementioned analysis accounted for 53% of the total phenotypic variation, with values ranging from 4.6 to 11.9%. Detailed information on these seven SNPs significantly associated with FER resistance across the two seasons and candidate genes is provided in Table 2. The candidate genes were identified using the B73 reference genome.

**Table 2:** Location, chromosome (Chr), *P*-values, position, and proportion of phenotypic variation explained (R^2^) for the most significant single nucleotide polymorphisms (SNPs) and corresponding candidate genes associated with fusarium ear rot resistance across the two seasons examined

SNP	Allele	Chr	Position	*P*-value	R^2^	Candidate gene	Annotation
2,396,181|F|0–39:G > T-39:G > T	G/T	1	210,459,200	0.000788	0.098	GRMZM2G104516	Zinc ion binding
2,463,074|F|0–20:G > A-20:G > A	G/A	4	181,707,160	0.0022	0.119	GRMZM2G047319	Serine-type endopeptidase activity
2,508,896|F|0–55:G > A-55:G > A	G/A	2	18,248,031	0.02137	0.053	GRMZM2G318949	Uncharacterized
2,591,766|F|0–50:A > G-50:A > G	A/G	2	22,241,420	0.00467	0.059	GRMZM2G337229	monolayer-surrounded lipid storage body
100,066,545|F|0–47:C > G-47:C > G	C/G	2	125,710,681	0.05477	0.046	GRMZM2G703158	Uncharacterized
2,383,629|F|0–19:T > C-19:T > C	T/C	5	12,219,628	0.0048	0.075	GRMZM2G068963	Protein peptidyl-prolyl isomerization
2,428,673|F|0–67:A > G-67:A > G	A/G	9	16,488,512	0.00712	0.081	GRMZM2G008152	uncharacterized

### Pathways

Pathway analysis identified seven significant pathways, namely LIPASYN-PWY, known as the phospholipase pathway, which hydrolyzes phospholipids and had nine genes involved in it contributing to the calculation of the enrichment score; PWY-5143, which is a fatty acid activation pathway and was associated with five genes; PWY-561, a superpathway of the glyoxylate cycle that links the conversion of fatty acids to carbohydrates and involved16 genes; PWY-5995, known as the linoleate biosynthesis I (plants) pathway and associated with eight genes; PWY-5136, a fatty acid β-oxidation II (plant peroxisome) pathway associated with nine genes; PWY-3561, a choline biosynthesis III pathway, which had the lowest number of genes associated with it (only four); and finally, PWY-5004, a superpathway of the citrulline metabolism, associated with nine genes (Table 3). Of the seven pathways, PWY-561 had the highest enrichment score (5.61) because the genes involved in it were among the highest ranking in the list. In contrast, PWY-3561 had the lowest enrichment score (2.06) and fewer high-ranking genes associated with it. LIPASYN-PWY, the phospholipase pathway, was the most significant (*p* = 0.01702). The details of these pathways, the genes involved, and their RESs are included in Table S1 and the associated graphs in Fig. S1.

**Table 3:** Summary of the gene set enrichment analysis for the seven most significant pathways detected

MaizeCyc ID	PW name	RES	*p*	Genes^a^	Significant gene ID
LIPASYN-PWY	phospholipases	3.97	0.017	9	GRMZM2G426556
PWY-3561	choline biosynthesis III	2.06	0.040	4	GRMZM2G133943
PWY-5004	superpathway of citrulline metabolism	4.26	0.044	9	GRMZM2G061990
PWY-5136	fatty acid &beta-oxidation II (core pathway)	3.76	0.033	9	GRMZM2G339336
PWY-5143	fatty acid activation	2.74	0.025	5	GRMZM2G339336
PWY-561	superpathway of glyoxylate cycle	5.61	0.027	16	GRMZM2G459755
PWY-5995	linoleate biosynthesis I (plants)	3.59	0.028	8	GRMZM2G339336

*PW* Pathway, *RES* Running enrichment score

^a^ The number of genes mapped to a pathway that contributes to the calculation of the enrichment score

*, **, *** Significant at the 0.05, 0.01, and 0.001 probability levels, respectively

## Discussion

Breeding for resistance is the best approach for managing FER, especially for smallholder farmers, who mostly grow maize for their own consumption and do not usually have the resources to adopt other control approaches (Chen et al. 2016). However, for an efficient use of this approach, it is important to identify sources of disease resistance that are effective and stable across environments. In this study, significant differences were detected among the 151 inbred lines evaluated for FER resistance across two seasons, and genotype-by-season interaction effects on FER resistance were also observed, as similarly reported in Afolabi et al. (2007) and Balconi et al. (2014).

The success of GWASs mainly depends on the LD of the genetic material examined because they exploit historical recombinations (de Jong et al. 2018; Kibe et al. 2020). In the present study, the rapid LD decay observed implied significant diversity in this panel of inbred lines, which made it suitable for GWAS (Kibe et al. 2020; Yan et al. 2011). Previous research has also reported rapid LD decay in tropical maize inbred lines (Romay et al. 2013; Coan et al. 2018; Kuki et al. 2020). LD is affected by both genetic and nongenetic factors, such as population stratification, genetic relatedness, recombination, linkage, genetic drift, selection, and mutation (Barreto et al. 2019; Flint-Garcia et al. 2003). The decay of LD is faster in tropical and subtropical lines since they are more genetically diverse and have more rare alleles than the temperate ones (Kuki et al. 2020).

GWASs have been successful in the genetic dissection of various complex traits. In this study, a GWAS was conducted to detect genomic regions and SNP markers associated with FER resistance in 151 tropical maize inbred lines. The comparison of the SNPs significantly associated with FER revealed no consistent marker-trait associations between the two seasons. Across seasons, seven significant SNPs were identified on chromosomes 1, 2, 4, 5, and 9. Each of these SNPs explained a small percentage of phenotypic variation, ranging from 4.6 to 11%, confirming that FER resistance is indeed a complex trait controlled by multiple QTLs with minor effects, in line with observations reported in previous research (Chen et al. 2016; Ju et al. 2017; Stagnati et al. 2019; Zila et al. 2013, 2014).

The candidate genes in this study were characterized as transcription factors as well as being involved in protein binding and intracellular signaling (Table 3). The GRMZM2G068963 gene on chromosome 5 is an FK506 binding protein. These proteins are known to play various roles in many processes critical for abiotic stress responses, plant growth, and development (Dong et al. 2018). *GRMZM2G104516* encodes for zinc finger proteins, which participate significantly in numerous biological processes, such as transcription, DNA recognition, translation, RNA packaging, regulation of apoptosis, protein–protein interaction, photosynthesis, lipid binding as well as in the regulation of resistance to various biotic (pathogen responses) and abiotic stresses (Ciftci-Yilmaz & Mittler 2008; Gupta et al. 2012; Laity et al. 2001; Stanton et al. 2022; Takatsuji 1998). *GRMZM2G337229*, also known as *ole1* or *oleosin1*, accounts for 80%–90% of the oil body structural proteins and plays an important role in lipid accumulation and storage (Chen et al. 2019). These results revealed the complexity of FER resistance in tropical maize and showed that various mechanisms may be involved in conditioning this resistance, including complex biosynthesis processes, which also may include interactions between numerous metabolic pathways (Chen et al. 2019).

It is important to note that the significant SNPs detected in this study differed from those identified in previous research. Chen et al. (2016) evaluated 818 tropical inbred lines using 43,424 SNP markers and identified 45 significant SNPs associated with FER resistance. In the present study, the nearest marker to those identified by Chen et al. (2016) was located on chromosome 5, at a distance of approximately 27 Mbp, and the same was observed by Guo et al. (2020), who identified 23 SNPs associated with FER resistance in a collection of 509 diverse inbred lines using 37,801 SNPs. Specifically, the study identified a SNP on chromosome 1 (position 226,233,417 bp) which was 15 Mbp away from the one detected in the present study at 210,459,200 bp. Another GWAS study conducted on a panel of 183 inbred lines using 267,525 SNP markers identified 14 SNPs significantly associated with FER resistance on chromosomes 1, 2, 3, 5, 6, 7, and 10 (Coan et al. 2018). In the present study, a SNP on chromosome 2 at position 22.24 Mbp was identified very close to the SNP on the same chromosome (9.6 Mbp).

According to Zila et al. (2014) and de Jong et al. (2018), these differences could be attributed to the different genetic backgrounds of the germplasm used, since the genetic background of populations significantly influences FER resistance, the markers used, the rapid LD decay, and the differences in sample sizes. The research confirmed that FER resistance is indeed a quantitative trait conditioned by many genes with relatively small effects that are not consistent between populations and environments (de Jong et al. 2018; Holland et al. 2020; Mesterházy et al. 2012; Zila et al. 2014).

Understanding the pathways involved in resistance is important to (1) advance our broader knowledge of plant defense mechanisms against pathogens (Tang et al. 2015), (2) complement conventional single-marker analysis in GWASs by providing necessary information, in particular through the identification of additional genes, and (3) elucidate the issue of “missing heritability” (Wang et al. 2010). Pathway analysis is also important in mechanistic research, as these reveal the underlying disease pathways without narrowing down each GWAS locus to a single gene (Wang et al. 2010). In this study, the most significant genes and their MaizeCyc enzyme annotations across the seven identified pathways were *GRMZM2G133943* (phosphatidate metabolism, as a signaling molecule), *GRMZM2G061990* (obsolete carbamoyl-phosphate synthase activity), *GRMZM2G426556* (no annotation), *GRMZM2G459755* (3-hydroxyacyl-CoA dehydrogenase), and *GRMZM2G339336*, which is significant for three MaizeCyc pathways (Table 3). Specifically, this gene was mapped to PWY-5136, PWY 5,143, and PWY-5995, and was located on chromosome 4 between positions 185,864,998 and 185,869,115 bp. *GRMZM2G339336* encodes a long-chain fatty acid-CoA ligase protein which plays a role in the physiological regulation of numerous cellular functions by producing long-chain fatty acyl-CoA esters. These, in turn, have been reported to be involved in protein transport, protein acylation enzyme activation, transcriptional regulation, and cell signaling (Fulda et al. 2002).

Among the detected pathways, the most significant one, LIPASYN-PWY, is regulated by a phospholipase that hydrolyzes phospholipids. Phospholipases are involved in various plant responses related to plant signal transduction, such as responding to auxin stimulation, pathogens, and elicitors (Ryu 2004), as well as responses to pathogen elicitation, abscisic acid, ethylene, nodulation, wounding, water loss, and seed germination (Wang & Wang 2001). According to Guo et al. (2009), pathway-based analysis is a useful and promising approach to effectively analyze GWAS data and detect disease variants by jointly considering gene variants that belong to the same biological pathway. For example, in this study, pathway PWY-561, which was the most significant, had 16 genes contributing to its enrichment score, implying that these genes were grouped together based on their shared biological functions, and their cumulative effects contributed to further elucidating the genetic differences that distinguished resistant and susceptible maize inbred lines (Tang et al. 2015). The results of this study are in line with those reported in Yao et al. (2020) indicating that FER resistance is a complex trait and depends on a network of multiple defense pathways.

For quantitative traits controlled by numerous genes, as in the case of FER resistance, the best strategy for molecular breeding is transitioning from marker-assisted breeding to genomic selection (Guo et al. 2020). In maize, genomic selection has been reported as an important genomics tool to improve breeding efficiency and accelerate genetic gain in several target traits, especially complex traits (Cao et al. 2021).

In conclusion, the creation of inbred maize lines resistant to FER or to the accumulation of the associated mycotoxin would be aided by the identification of the pertinent alleles and metabolites involved in the resistance mechanism. The present study contributed to such knowledge by identifying significant genes via GWAS and pathway analysis. It is advisable to use pathway analysis in conjunction with GWASs to (1) identify biological processes that are broadly distributed across an entire network of genes that have subtle effects at the individual level and (2) detect biological aspects that could have been missed while concentrating on only one or a few genes exhibiting the most significant associations with the trait of interest. FER-resistant inbred lines could also potentially be used as sources to develop hybrids resistant to this disease.

### Supplementary Information

Below is the link to the electronic supplementary material.

Supplementary file1 (DOCX 274 kb)
