# Supplementary material for: Genome-wide association study and pathway analysis to decipher loci associated with Fusarium ear rot resistance in tropical maize germplasm
Source: Genet Resour Crop Evol. 2023 Nov 10;71(6):2435–48. doi: 10.1007/s10722-023-01793-4 (PMC11252232; doi:10.1007/s10722-023-01793-4)
Supplement: Supplementary file 1 — Supplementary file1 (DOCX 274 kb) [file 10722_2023_1793_MOESM1_ESM.docx]

Supplementary Material

Genome-Wide Association, and Pathway Analysis Study to Decipher Loci Associated with Fusarium Ear Rot Resistance in Tropical Maize Germplasm

Stella Bigirwa Ayesiga^1 3^, Patrick Rubaihayo^1^, Bonny Michael Oloka^2^, Isaac Onziga Dramadri^1^, and Julius Pyton Sserumaga^3*^

*** Correspondence:** Julius Pyton Sserumaga**:** j.serumaga@gmail.com


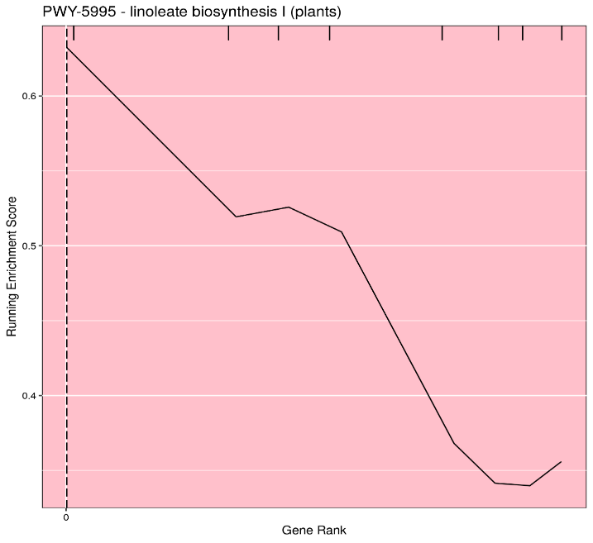

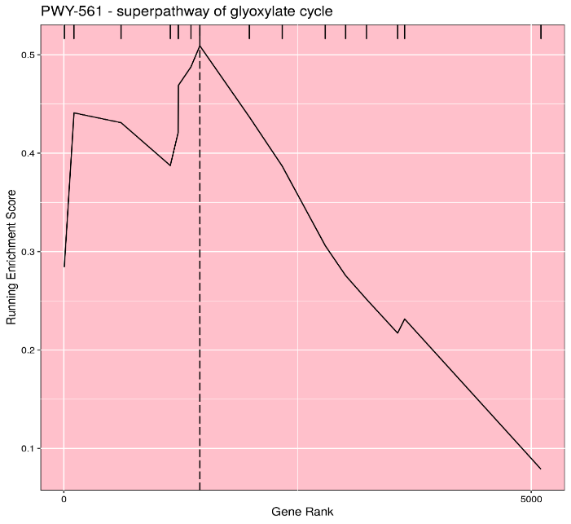


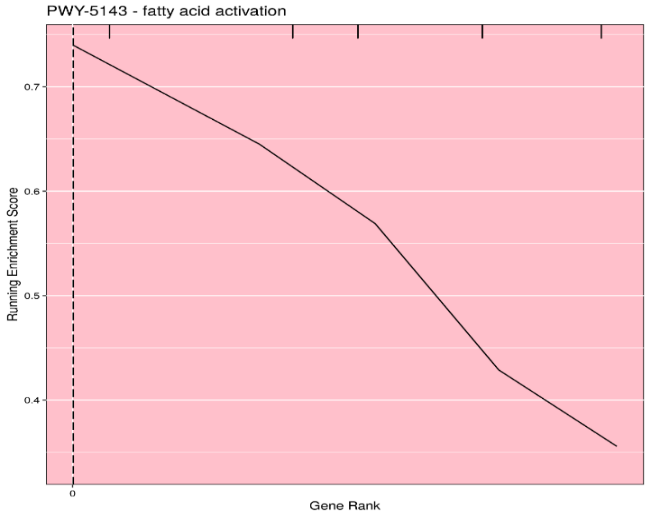

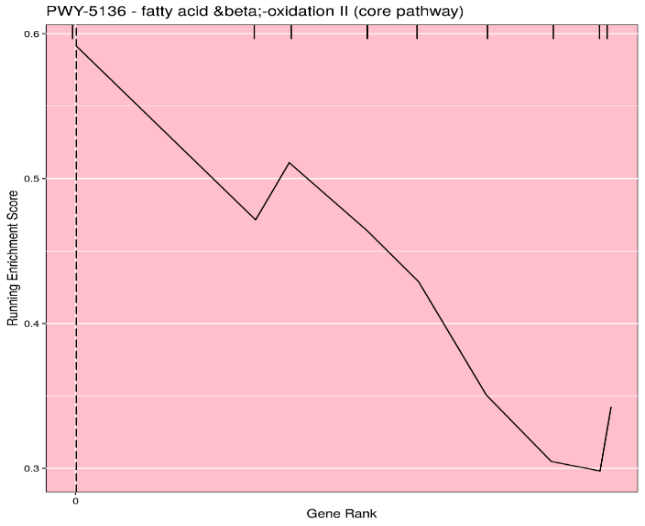


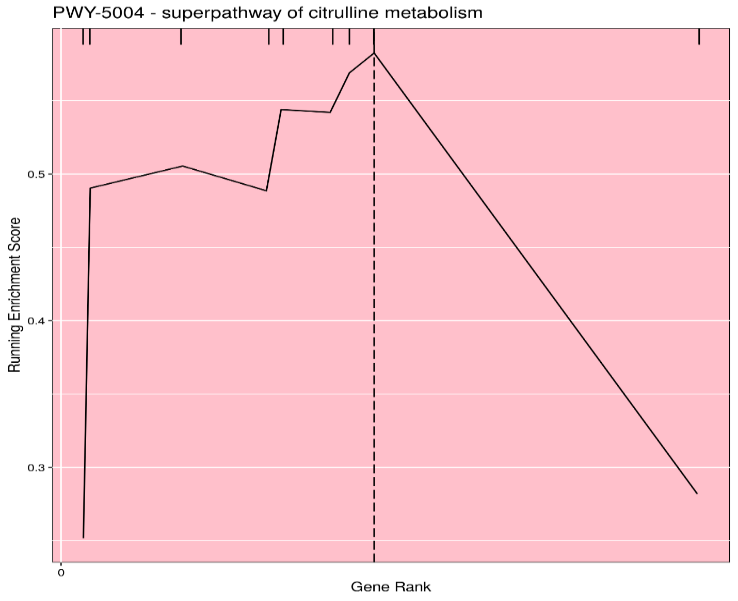

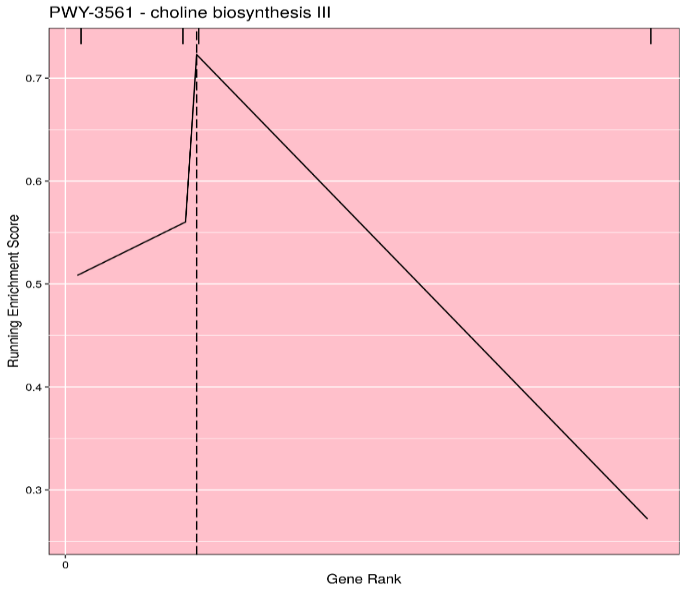


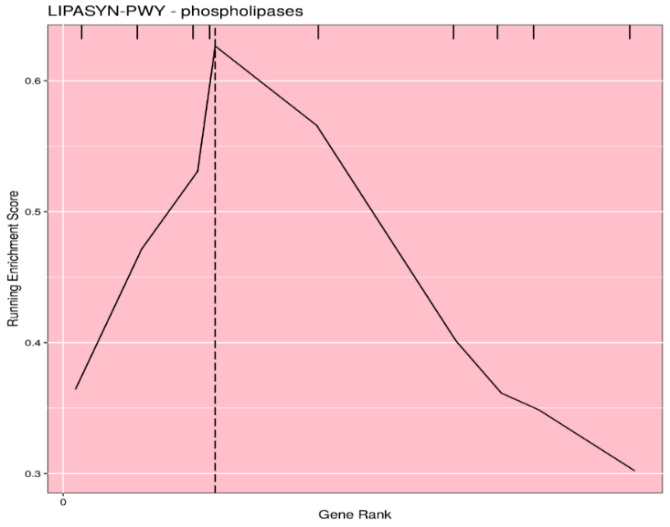


*Genes were ranked in ascending order by their effect scores, with hash marks at the top of the graph signifying the ranks of genes in the respective pathway. The pathway enrichment score which coincided with the maximum running enrichment score is marked by the dashed vertical line

**Figure S1.** Graphs of the RES for the seven pathways.

**Table S1.** Summary of the gene-set enrichment analysis for pathways

| **PW ID** | **PW No.** | **gene_id** | **RES** | **pathway_name** | **p-value** |  |
| --- | --- | --- | --- | --- | --- | --- |
| LIPASYN-PWY | 1 | GRMZM2G108912 | 0.364299 | phospholipases | 0.017002 |  |
| LIPASYN-PWY | 1 | GRMZM2G318860 | 0.47141 | phospholipases | 0.017002 |  |
| LIPASYN-PWY | 1 | GRMZM2G133943 | 0.530859 | phospholipases | 0.017002 |  |
| LIPASYN-PWY | 1 | GRMZM2G426556 | 0.62653 | phospholipases | 0.017002 |  |
| LIPASYN-PWY | 1 | GRMZM2G114354 | 0.565908 | phospholipases | 0.017002 |  |
| LIPASYN-PWY | 1 | GRMZM2G406951 | 0.401495 | phospholipases | 0.017002 |  |
| LIPASYN-PWY | 1 | GRMZM2G090230 | 0.361469 | phospholipases | 0.017002 |  |
| LIPASYN-PWY | 1 | GRMZM2G023335 | 0.348645 | phospholipases | 0.017002 |  |
| LIPASYN-PWY | 1 | GRMZM2G045294 | 0.302115 | phospholipases | 0.017002 |  |
| PWY-5143 | 2 | GRMZM2G339336 | 0.73975 | fatty acid activation | 0.025497 |  |
| PWY-5143 | 2 | GRMZM2G118286 | 0.645052 | fatty acid activation | 0.025497 |  |
| PWY-5143 | 2 | GRMZM2G145179 | 0.568952 | fatty acid activation | 0.025497 |  |
| PWY-5143 | 2 | GRMZM2G104847 | 0.428791 | fatty acid activation | 0.025497 |  |
| PWY-5143 | 2 | GRMZM2G079236 | 0.355736 | fatty acid activation | 0.025497 |  |
| PWY-561 | 3 | GRMZM2G339336 | 0.284189 | superpathway of glyoxylate cycle | 0.026732 |  |
| PWY-561 | 3 | GRMZM2G063851 | 0.440874 | superpathway of glyoxylate cycle | 0.026732 |  |
| PWY-561 | 3 | GRMZM2G580389 | 0.430932 | superpathway of glyoxylate cycle | 0.026732 |  |
| PWY-561 | 3 | GRMZM2G010823 | 0.387371 | superpathway of glyoxylate cycle | 0.026732 |  |
| PWY-561 | 3 | GRMZM2G164714 | 0.420874 | superpathway of glyoxylate cycle | 0.026732 |  |
| PWY-561 | 3 | GRMZM2G118286 | 0.468885 | superpathway of glyoxylate cycle | 0.026732 |  |
| PWY-561 | 3 | GRMZM2G064023 | 0.487413 | superpathway of glyoxylate cycle | 0.026732 |  |
| PWY-561 | 3 | GRMZM2G459755 | 0.509039 | superpathway of glyoxylate cycle | 0.026732 |  |
| PWY-561 | 3 | GRMZM2G145179 | 0.436575 | superpathway of glyoxylate cycle | 0.026732 |  |
| PWY-561 | 3 | GRMZM2G398500 | 0.386828 | superpathway of glyoxylate cycle | 0.026732 |  |
| PWY-561 | 3 | GRMZM2G104847 | 0.305933 | superpathway of glyoxylate cycle | 0.026732 |  |
| PWY-561 | 3 | GRMZM2G001696 | 0.275652 | superpathway of glyoxylate cycle | 0.026732 |  |
| PWY-561 | 3 | GRMZM2G117357 | 0.251425 | superpathway of glyoxylate cycle | 0.026732 |  |
| PWY-561 | 3 | GRMZM2G079236 | 0.217131 | superpathway of glyoxylate cycle | 0.026732 |  |
| PWY-561 | 3 | GRMZM2G177404 | 0.231452 | superpathway of glyoxylate cycle | 0.026732 |  |
| PWY-561 | 3 | GRMZM2G119950 | 0.078928 | superpathway of glyoxylate cycle | 0.026732 |  |
| PWY-5995 | 4 | GRMZM2G339336 | 0.632342 | linoleate biosynthesis I (plants) | 0.02764 |  |
| PWY-5995 | 4 | GRMZM2G118286 | 0.519289 | linoleate biosynthesis I (plants) | 0.02764 |  |
| PWY-5995 | 4 | GRMZM2G064701 | 0.525745 | linoleate biosynthesis I (plants) | 0.02764 |  |
| PWY-5995 | 4 | GRMZM2G145179 | 0.509229 | linoleate biosynthesis I (plants) | 0.02764 |  |
| PWY-5995 | 4 | GRMZM2G104847 | 0.368092 | linoleate biosynthesis I (plants) | 0.02764 |  |
| PWY-5995 | 4 | GRMZM2G161792 | 0.34148 | linoleate biosynthesis I (plants) | 0.02764 |  |
| PWY-5995 | 4 | GRMZM2G056252 | 0.339841 | linoleate biosynthesis I (plants) | 0.02764 |  |
| PWY-5995 | 4 | GRMZM2G079236 | 0.355929 | linoleate biosynthesis I (plants) | 0.02764 |  |
| PWY-5136 | 5 | GRMZM2G339336 | 0.59153 | fatty acid &beta;-oxidation II (core pathway) | 0.03252 |  |
| PWY-5136 | 5 | GRMZM2G118286 | 0.471508 | fatty acid &beta;-oxidation II (core pathway) | 0.03252 |  |
| PWY-5136 | 5 | GRMZM2G459755 | 0.510988 | fatty acid &beta;-oxidation II (core pathway) | 0.03252 |  |
| PWY-5136 | 5 | GRMZM2G145179 | 0.463977 | fatty acid &beta;-oxidation II (core pathway) | 0.03252 |  |
| PWY-5136 | 5 | GRMZM2G398500 | 0.428799 | fatty acid &beta;-oxidation II (core pathway) | 0.03252 |  |
| PWY-5136 | 5 | GRMZM2G104847 | 0.350574 | fatty acid &beta;-oxidation II (core pathway) | 0.03252 |  |
| PWY-5136 | 5 | GRMZM2G117357 | 0.304772 | fatty acid &beta;-oxidation II (core pathway) | 0.03252 |  |
| PWY-5136 | 5 | GRMZM2G079236 | 0.298198 | fatty acid &beta;-oxidation II (core pathway) | 0.03252 |  |
| PWY-5136 | 5 | GRMZM2G177404 | 0.342434 | fatty acid &beta;-oxidation II (core pathway) | 0.03252 |  |
| PWY-3561 | 6 | GRMZM2G108912 | 0.508387 | choline biosynthesis III | 0.040397 |  |
| PWY-3561 | 6 | GRMZM2G132898 | 0.560269 | choline biosynthesis III | 0.040397 |  |
| PWY-3561 | 6 | GRMZM2G133943 | 0.722895 | choline biosynthesis III | 0.040397 |  |
| PWY-3561 | 6 | GRMZM2G466281 | 0.271676 | choline biosynthesis III | 0.040397 |  |
| PWY-5004 | 7 | GRMZM2G130440 | 0.251771 | superpathway of citrulline metabolism | 0.044367 |  |
| PWY-5004 | 7 | GRMZM2G700683 | 0.490432 | superpathway of citrulline metabolism | 0.044367 |  |
| PWY-5004 | 7 | GRMZM2G124321 | 0.505474 | superpathway of citrulline metabolism | 0.044367 |  |
| PWY-5004 | 7 | GRMZM2G047208 | 0.48861 | superpathway of citrulline metabolism | 0.044367 |  |
| PWY-5004 | 7 | GRMZM2G028535 | 0.543937 | superpathway of citrulline metabolism | 0.044367 |  |
| PWY-5004 | 7 | GRMZM2G007953 | 0.542111 | superpathway of citrulline metabolism | 0.044367 |  |
| PWY-5004 | 7 | GRMZM2G170013 | 0.568986 | superpathway of citrulline metabolism | 0.044367 |  |
| PWY-5004 | 7 | GRMZM2G061990 | 0.582544 | superpathway of citrulline metabolism | 0.044367 |  |
| PWY-5004 | 7 | GRMZM2G339523 | 0.281866 | superpathway of citrulline metabolism | 0.044367 |  |

*ID identifier, PW pathway, RES running enrichment score
